# COVID-19 pandemic-driven evolution of *Klebsiella pneumoniae* : rising resistance, genetic diversity, and virulence in a Brazilian tertiary hospital

**DOI:** 10.31744/einstein_journal/2026AO1627

**Published:** 2026-03-30

**Authors:** Romário Oliveira de Sales, Laura Leaden, Paula Célia Mariko Koga, André Mario Doi, Letícia Busato Migliorini, Alexandra do Rosario Toniolo, Fernando Gatti de Menezes, Marines Dalla Valle Martino, Patricia Severino

**Affiliations:** 1 Instituto Israelita de Ensino e Pesquisa Albert Einstein Hospital Israelita Albert Einstein São Paulo SP Brazil Instituto Israelita de Ensino e Pesquisa Albert Einstein, Hospital Israelita Albert Einstein, São Paulo, SP, Brazil.; 2 Laboratório Clínico Hospital Israelita Albert Einstein São Paulo SP Brazil Laboratório Clínico, Hospital Israelita Albert Einstein, São Paulo, SP, Brazil.; 3 Serviço de Controle de Infecção Hospitalar Hospital Israelita Albert Einstein São Paulo SP Brazil Serviço de Controle de Infecção Hospitalar, Hospital Israelita Albert Einstein, São Paulo, SP, Brazil.

**Keywords:** Klebsiella pneumoniae, COVID-19, cgMLST, Plasmids, Virulence, Infections, Colonization

## Abstract

**Objective:**

Coronavirus disease 2019 (COVID-19) caused increased intensive care unit admissions, invasive procedures, and antimicrobial use, potentially worsening bacterial infections and multidrug resistance. Retrospective studies have found that *Klebsiella pneumoniae* co-infections in COVID-19 patients were significant and possibly linked to mechanical ventilation and central catheter placement. In the present study, we assessed the genetic diversity and antimicrobial resistance of *K. pneumoniae* isolates collected before and during the pandemic to evaluate the effect of the pandemic on these factors.

**Methods:**

Antimicrobial resistance profiling, whole-genome sequencing, and phylogenetic analyses were used to examine resistance patterns, genetic diversity, and mobile genetic elements.

**Results:**

From January 2018 to January 2021, 263 *K. pneumoniae* isolates were identified from infection sites. Carbapenem-resistant isolates increased from 32.5% in 2019 to 60.3% in 2020, remaining stable until January 2021. Nevertheless, healthcare-associated infections did not increase significantly, highlighting the effectiveness of infection control programs. Whole-genome sequencing showed that 54% of carbapenem-resistant isolates carried plasmids resembling pKPC_FCF3SP (IncN) and pKpQIL-like (IncFII) plasmids; however, the number of pKpQIL-like plasmid carriers declined during the pandemic likely due to patient transfer carrying isolates with distinct mobilome. Simultaneously, the number of plasmid-negative isolates increased by 25%. Carbapenem-resistant isolates showed multidrug resistance, particularly to cephalosporins and fluoroquinolones; however, aminoglycosides remained effective. Genetic analysis identified ten aminoglycoside resistance genes, with *aac(6′)-Ib-D181Y* associated with improved substrate recognition, being the most prevalent. Virulence factors included four integrative conjugative elements, with the integrative conjugative element *K. pneumoniae* ICEKp4 found only in pandemic period isolates. The O4 and O2 antigens predominated, whereas O3b appeared exclusively during the pandemic.

**Conclusion:**

The COVID-19 pandemic has contributed to a rise in carbapenem-resistant *K. pneumoniae* , underscoring the need for ongoing surveillance. Molecular shifts reflect the adaptation of the pathogen to evolving clinical settings.

## HIGHLIGHTS

Carbapenem resistance rose sharply during the COVID-2019 pandemic, doubling from 2019 to 2020, while infection control practices prevented proportional increases in healthcare-associated infections.

Genomic analyses revealed high diversity among *bla*_KPC_-positive isolates, with an expansion of sequence types and a predominance of sporadic non-outbreak clones throughout the pandemic period.

Shifts in mobile genetic elements and virulence markers occurred during the pandemic, including the emergence of the integrative and conjugative element of *Klebsiella pneumoniae* ICEKp4, changes in O-antigen types, and reduced prevalence of pKpQIL-like plasmids.

## In Brief

During the COVID-2019 pandemic, carbapenem-resistant *Klebsiella pneumoniae* increased and showed broader genetic diversity, new mobile elements, and changing plasmid profiles. Most isolates were sporadic, highlighting the importance of genomic surveillance and effective infection control practices.

## Graphical abstract

**Figure:**
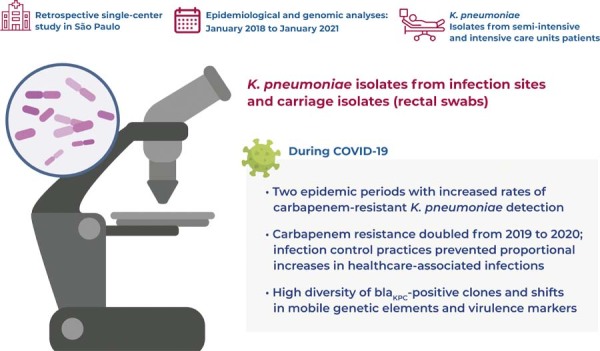

## INTRODUCTION

In 2019, severe acute respiratory syndrome coronavirus 2 (SARS-CoV-2), which causes coronavirus disease 2019 (COVID-19), was identified in China and spread rapidly worldwide.^( 1 )^ The first COVID-19 case in Brazil was recorded on February 25, 2020.^( 2 )^ Since then, COVID-19 has been associated with 714,534 deaths (according to https://infoms.saude.gov.br/extensions/covid-19_html/ covid-19_html.html consulted on January 16, 2025). During the COVID-19 pandemic, hospitals became overcrowded, with some medical centers reporting 50% more patients than usual.^( 2 )^ The exponential rise in admissions to intensive care units (ICUs), extended patient stays, and increased use of invasive procedures and immunosuppressive drugs may have contributed to an increase in bacterial infections among patients admitted with COVID-19.^( 2 , 3 )^

*Klebsiella pneumoniae* is one of the most common Gram-negative pathogens associated with hospital- and community-acquired infections. *K. pneumoniae* is an asymptomatic colonizer of the human gastrointestinal tract and is associated with clinical infections in patients admitted to ICUs.^( 4 - 6 )^ Resistance to carbapenems in Enterobacterales is mostly associated with horizontally acquired beta-lactamases such as *K. pneumoniae* carbapenemases (KPC) and metallo-beta-lactamases (NDM, IMP, and VIM).^( 4 , 7 )^ Other genes such as *fosA* , *oqxAB* , and *aac(6′)-Ib* , conferring resistance to fosfomycin, quinolones, and aminoglycosides, respectively, are also horizontally acquired by *K. pneumoniae* .^( 8 )^ In Brazil, *K. pneumoniae* CC258 carrying KPC is endemic, with sequence types (ST) ST11, ST258, ST512, and ST437 the most prevalent.^( 9 - 12 )^

Bacterial coinfection is associated with an increased risk of morbidity and mortality in viral infections.^( 13 )^ Secondary infection with *K. pneumoniae* has been associated with severe respiratory complications in ICU patients.^( 4 )^ Retrospective studies in patients with SARS-CoV-2 found coinfection with *K. pneumoniae* in 22–55% of cases.^( 13 - 15 )^ Additionally, the most common resistance gene carried by *K. pneumoniae* was KPC, followed by OXY-48, CTX-M, TEM, NDM, and SHV.^( 4 )^ These infections may be associated with invasive mechanical ventilation and central catheter placement.^( 4 )^

During the COVID-19 pandemic, use of broad-spectrum antibiotics like carbapenems rose sharply in ICU patients with COVID-19 due to uncertainty about bacterial co-infection.^( 4 , 16 )^ Reports show that 72% of hospitalized COVID-19 patients received antibiotics, although only 54% had a suspected or confirmed bacterial infection.^( 17 )^ This may have promoted the selection and spread of resistant pathogens,^( 4 )^ posing a public health concern.^( 18 , 19 )^

In this study, we investigated whether the onset of the COVID-19 pandemic influenced the population structure, antimicrobial resistance profiles, and virulence gene content of KPC-producing *K. pneumoniae* isolates from patients at a tertiary care hospital in Brazil. We studied the changes in the population of KPC-harboring *K. pneumoniae* isolates, as compared with our previous reports,^( 9 , 11 )^ after the first case of COVID-19 recorded on February 25, 2019, in this hospital. The results are presented in terms of pulsed-field gel electrophoresis (PFGE) types, multilocus sequence typing (MLST), antimicrobial susceptibility, presence of virulence genes, and plasmid content.

## OBJECTIVE

To assess whether the onset of the COVID-19 pandemic altered the population structure, antimicrobial resistance profiles, and virulence gene content of KPC-producing *K. pneumoniae* isolates in a Brazilian tertiary hospital, post-pandemic isolates were compared with previously characterized collections using pulsed-field gel electrophoresis, multilocus sequence typing, antimicrobial susceptibility testing, virulence gene screening, and plasmid analysis.

## METHODS

### Selection of isolates and determination of susceptibility markers for endemic level graph construction

The isolates evaluated for *K. pneumoniae* were identified by the Microbiology Department of the Clinical Laboratory at *Hospital Israelita Albert Einstein* between January 2018 and January 2021. The selected isolates were obtained from patients admitted to the ICU or semi-ICU. Isolates from blood, bronchoalveolar lavage, and tracheal secretions were considered infection-associated isolates for the purpose of this study, and isolates from rectal swabs as part of the institutional multidrug-resistant bacteria surveillance program were carriage isolates. When an isolate of infection or colonization was reported from the same patient with the same collection date, isolation site, and susceptibility profile, only the first report of the isolate was retained for further analysis.

Species confirmation of *K. pneumoniae* was performed using matrix-assisted laser desorption/ionization time-of-flight mass spectrometry (MALDI-TOF MS) (Bruker Daltonics, Billerica, MA, USA). Antimicrobial profile evaluation was conducted using the disk diffusion method for imipenem and meropenem in surveillance cultures (colonization samples) and the automated VITEK 2 system (bioMérieux, France) for infection samples. The VITEK 2 system includes the following antimicrobials: ceftazidime, cefepime, amikacin, gentamicin, ciprofloxacin, imipenem, and meropenem, with carbapenem resistance confirmed using gradient diffusion methods. The antimicrobial susceptibility results were interpreted according to the Brazilian Committee on Antimicrobial Susceptibility Testing/European Committee on Antimicrobial Susceptibility Testing guidelines for each year (http://brcast.org.br/). The proportion of resistant isolates was calculated by dividing the number of resistant *K. pneumoniae* isolates by the total number of isolates tested against the corresponding antibiotics and multiplying by 100.^( 20 )^ The endemic level of *K. pneumoniae* isolates was calculated per 10,000 patient-days, according to the method described by Arantes et al.^( 21 )^ The presence of the *bla*_KPC_ gene in isolates showing reduced susceptibility to carbapenems was determined by real-time polymerase chain reaction (PCR).^( 22 )^

To characterize the population of *K. pneumoniae* carrying *bla*_KPC_ (KpKPC) circulating in the hospital while avoiding community-acquired isolates, molecular analyses were restricted to KpKPC from colonized patients or those with infections admitted to the ICU or semi-ICU during the periods of highest KpKPC prevalence and adjacent periods between January 2018 and January 2021. Furthermore, bacterial isolates for molecular analyses were selected under two conditions: availability of isolates in the isolate bank of the Clinical Laboratory and bacterial viability. Only one isolate per patient was included in the analysis, corresponding to the first colonization and/or infection sample positive for KpKPC.

### Pulsed-field gel electrophoresis

Genetic relatedness was established using PFGE as described previously.^( 23 )^ For the PFGE result interpretation, the Dice similarity coefficient was used, and isolates were considered identical when patterns showed ≥90% similarity.^( 24 )^ Isolates were classified as endemic or sporadic following Riley et al.^( 25 )^ Dendrograms were generated using BioNumerics v7.5 for the visualization of PFGE results.

### Whole genome sequencing

Whole genome sequencing (WGS) was performed according to the protocol described by Migliorini et al.^( 11 )^ *De novo* assembly was used to determine STs through the software ‘mlst’ v2.19.0 with default settings (https://github.com/tseemann/mlst, accessed on October 20, 2021), using the *K. pneumoniae* scheme available via Institut Pasteur’s database (https://bigsdb.pasteur.fr/). Identification of capsule synthesis *loci* (K-loci or KL) and O antigen (lipopolysaccharide) serotype prediction were performed using Kleborate v.2.2.0 with default settings.^( 26 )^ Sequence assemblies were annotated using the Prokka v1.14.6 bacterial annotation pipeline with default parameters.^( 27 )^ Annotated GFF3 files were used for core genome definition using Roary v3.13.0, choosing a minimum BLASTP identity of 95% and a core gene prevalence of >99% in all isolates. The core genome alignment performed by Roary was used to infer a maximum-likelihood phylogeny using IQ-TREE v2.2.0 with 1000 ultrafast bootstrap replicates.^( 28 )^ IQ-TREE was used with the ModelFinder module, which allows the selection of the best nucleotide substitution model based on the input data. For our dataset, the best model was GTR+F+I+I+R2. The phylogenetic tree was visualized using iToL software.^( 29 )^ The clonality and relatedness of the isolates were determined using the core-genome MLST (cgMLST) analysis implemented in the chewBBACA pipeline.^( 30 )^ Briefly, the gene prediction algorithm, Prodigal, was trained using *K. pneumoniae* subsp. *pneumoniae* NTUH-K2044 (accession number: NC_012731.1). A reference cgMLST dataset for the species complex *K. pneumoniae* , *K. variicola* , and *K. quasipneumoniae* was downloaded from https://www.cgmlst.org/ and used for cgMLST. The resulting cgMLST data were analyzed using GrapeTree v1.5.0 to obtain a minimum spanning tree with parameters implemented in MSTree v2, ignoring missing values for the entire collection.^( 31 )^

The resistome, virulome, and incompatibility groups were analyzed using ABRicate v.1.0.1 (ABRicate, https://github.com/tseemann/abricate). Using this tool, a blastn search of genes included in the NCBI AMRFinderPlus, VFDB, and PlasmidFinder databases was performed on the *de novo* whole-genome assembly.^( 32 - 34 )^ The presence of the integrative and conjugative element of *K. pneumoniae* (ICEKp) was identified using Kleborate v.2.2.0 with default settings.^( 26 )^

This work was approved by the Institutional Review Board of *Hospital Israelita Albert Einstein* (CAAE: 39720720.7.0000.0071; # 4.404.677).

## RESULTS

### Characterization of the isolates and epidemiological analysis

**Figure 1:**
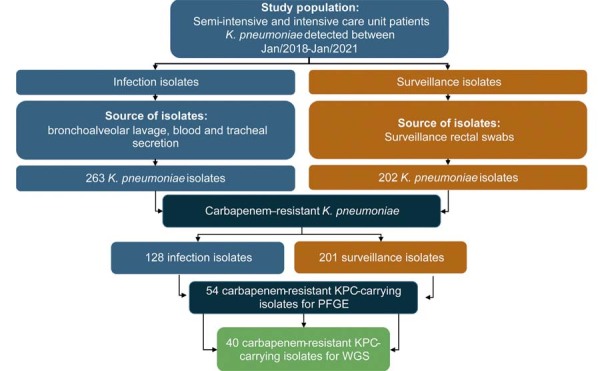
Flowchart describing the sample selection procedures used in this study. The bacterial isolates for molecular analyses were selected based on their availability in the Clinical Laboratory biobank and their bacterial viability. At least one isolate per pulsotype was subjected to whole-genome sequencing.

Between January 2018 and January 2021, 263 *K. pneumoniae* isolates were identified from the infection sites (blood, bronchoalveolar lavage, and tracheal secretions) of patients admitted to the ICU and semi-ICU, and 202 isolates of *K. pneumoniae* were identified in surveillance rectal swabs. The 263 isolates were distributed among the following infection sites: tracheal secretions (181 isolates, 68.8%), blood (56 isolates, 21.3%), and bronchoalveolar lavage (26 isolates, 9.9%). Of these isolates, 51.3% were susceptible to carbapenems, referred to here as non-KpCR, and the remaining isolates were carbapenem-resistant, referred to in this study as KpCR. Figure 1 summarizes the study population and filters used for the selection of isolates for molecular analyses.

PFGE: pulsed-field gel electrophoresis. WGS: whole-genome sequencing. KPC: Klebsiella pneumoniae carbapenemase.

**Figure 2:**
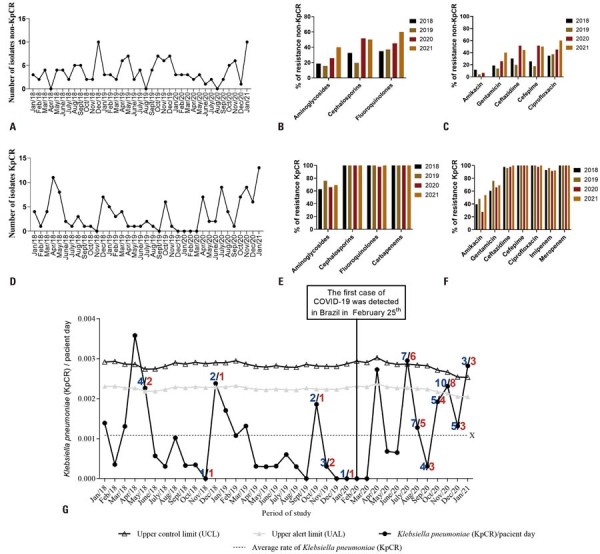
(A) Distribution of the 135 *Klebsiella pneumoniae* isolates considered to be non-resistant to carbapenem (non-KpCR) from January 2018 to January 2021. (B) Resistance profile by antimicrobial class for the 135 non-KpCR isolates. (C) Resistance profile by antimicrobial for the 135 non-KpCR isolates. (D) Distribution of the KpCR isolates reported from January 2018 to January 2021. (E) Resistance profile by antimicrobial class for the 128 KpCR isolates. (F) Resistance profile by antimicrobial for the 128 KpCR isolates. Classes: aminoglycosides ( *amikacin* and *gentamicin* ), cephalosporins ( *cefepime* and *ceftazidime* ), fluoroquinolones ( *ciprofloxacin* ), and carbapenems ( *imipenem* and *meropenem* ). (G) Temporal distribution of KpCR isolates reported in intensive care and semi-intensive care units per patient day from January 2018 to January 2021. X: average rate of isolates for the analyzed period (average KpCR infection rate: 0.001080552 or 10.80 per 10,000 patient days). The highlighted numbers above the variable *Rates KpCR/patient day* represent the number of isolates subjected to pulsed-field gel electrophoresis in blue and the number of isolates subjected to whole genome sequencing in red (see section *Molecular epidemiology and comparative genomics* ).

For the four classes of antimicrobials tested against the non-KpCR isolates, we observed an increase in the percentage of resistant isolates from 2020 to 2021 compared to that in the 2018–2019 period ( Figure 2A and 2B). A similar trend was observed for each antimicrobial, except for amikacin, which showed the highest resistance rates in 2018 and 2021 (11.6% and 6.4%, respectively) and the lowest rates in 2019 and 2020 (3.8% and 0%, respectively). Notably, in January 2021, the last period evaluated in this study, 10 isolates were tested for amikacin, and none were resistant ( Figure 2C ). Thus, for the non-KpCR isolates, the most effective antimicrobial *in vitro* was amikacin, with an average susceptibility of 94.5% over the entire study period ( Figure 2B ). Meanwhile, 48.7% (128/263) of the isolates were resistant to carbapenems (imipenem and/or meropenem), referred to as KpCR. The highest number of reported KpCR isolates was 13 in January 2021 ( Figure 2D ). These isolates also exhibited high resistance to the other classes of antimicrobials evaluated in this study (Figures 2E and 2F). The analysis of isolates from surveillance rectal swabs revealed that 99.5% (201/202) were resistant to carbapenems (imipenem and/or meropenem).

Between January 2018 and January 2021, the average hospital-acquired infection (HI) rate associated with KpCR was 10.80 per 10,000 patient days. During this period, three instances exceeded the established control limit (April 2018, July 2020, and January 2021), indicating an epidemic period, with hospital infection rates of 35.8, 29.5, and 28.2, respectively ( Figure 2G ). During the pandemic, there were two instances (July 2020 and January 2021) in which the HI rate for KpCR exceeded the established control limit (29.5 and 28.2, respectively); however, these rates were lower than those in April 2018 before the pandemic ( Figure 2G ).

### Molecular epidemiology and comparative genomics

A total of 54 KpKPC isolates were analyzed using PFGE following the selection criteria outlined in the Methods section. These isolates were distributed across 13 points on the endemic level graph ( Figure 2G , highlighted in blue) and classified as either infection isolates (14/54; 25.9%) or colonization isolates (40/54; 74%).

**Figure 3:**
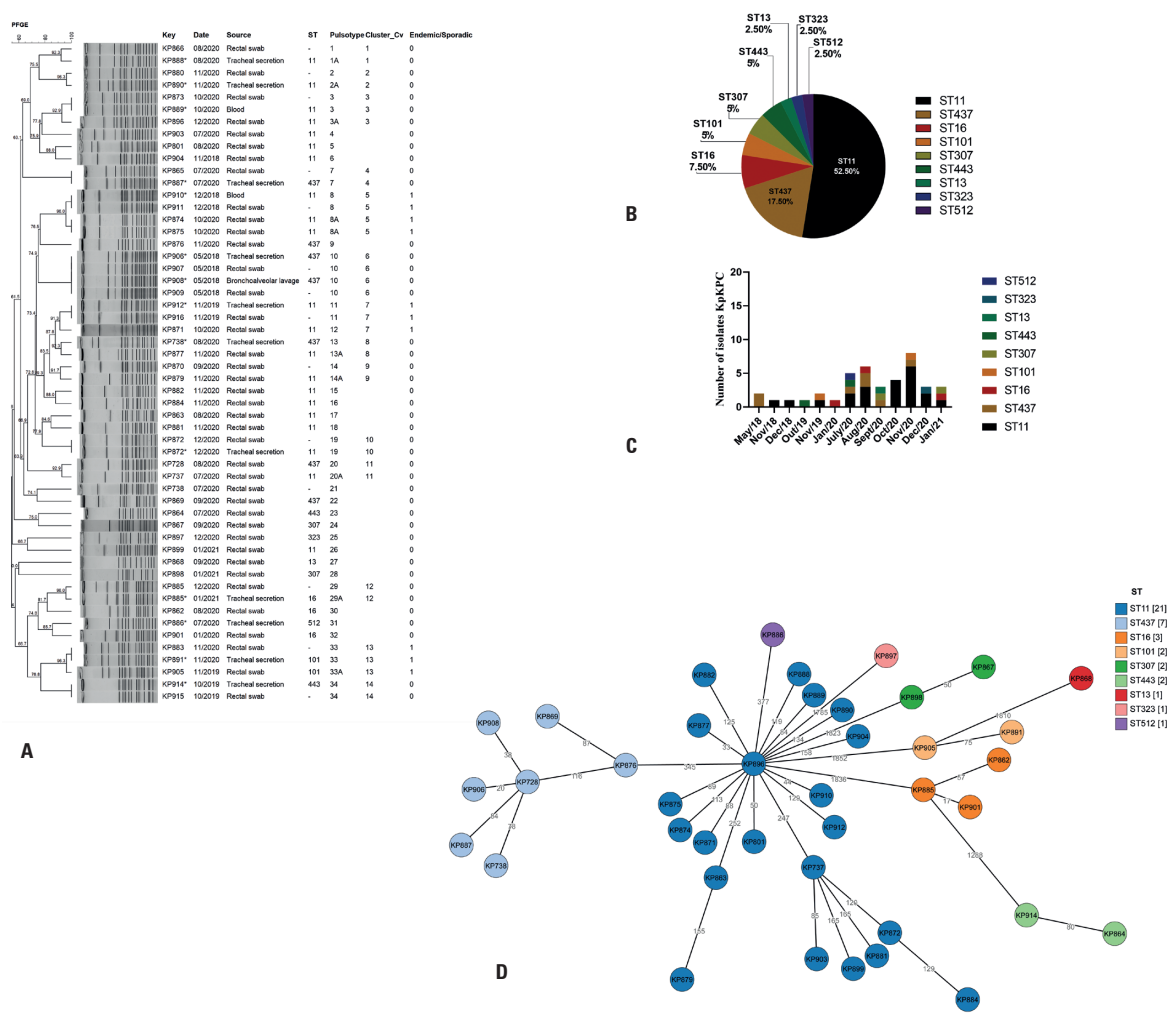
(A) Dendrogram showing the relationship of the 54 *Klebsiella pneumoniae* isolates carrying *bla* KPC (KpKPC) based on pulsed-field gel electrophoresis (PFGE) results. Key: isolate identification, Date: date of isolation (month/year). Source: body site, ST: sequence type, Cluster: two or more isolates share >90% similarity, Endemic and Sporadic: (0) sporadic and (1) endemic. Pulsotype: identification attributed to the PFGE band pattern for the purpose of this study. Infection isolates sharing the same identification number as colonization isolates. (B) Pie chart showing the prevalence of each ST during the whole studied period. (C) ST identification per month and year. (D) Minimum spanning tree showing the genetic relatedness of the 40 isolates in this study, determined from core-genome multilocus sequence typing (cgMLST) data. Isolates are color-coded by ST.

Pulsed-field gel electrophoresis analysis of these 54 KpKPC isolates identified 34 distinct pulsotypes, forming 14 clusters (defined as two or more isolates with ≥90% similarity), grouping 64.8% of KpKPC isolates ( Figure 3A ). Clusters 8, 9, 11, 16, 18, and 33 included endemic KpKPC isolates, accounting for 22.2% (12/54), whereas the remaining 77.8% (42/54) were classified as sporadic. Among the infection isolates (14/54; 25.9%), 21.4% (3/14) were endemic, and 66.7% (2/3) were identified after the first COVID-19 case in Brazil. Similarly, 22.5% (9/40) of the carriage isolates obtained from surveillance rectal swabs were endemic, and 66.7% (6/9) of these were identified after the first COVID-19 case. These findings indicate that the proportion of endemic isolates remained consistent before and during the COVID-19 pandemic.

Specific KpKPC isolates were selected for PFGE typing during the periods when the established control limit for KpCR was exceeded following the first confirmed COVID-19 case in Brazil (July 2020 and January 2021) ( Figure 2G ). In the first period (July 2020), seven isolates were analyzed, of which five had different pulsotypes and were classified as sporadic clones. In the second period (January 2021), three isolates were analyzed, all with different pulsotypes and classified as sporadic clones. These results suggest an increase in sporadic KpKPC isolates during epidemic periods, when the control limit for KpCR was exceeded.

Between May 2018, when the earliest KpKPC isolate was analyzed by PFGE, and January 2020, the last month before COVID-19 reached Brazil, 13 isolates were typed, with 38.5% (5/13) classified as endemic and 61.5% (8/13) as sporadic. From February 25, 2020, when the first COVID-19 case was reported in Brazil, until January 2021, the last month of analysis, 41 KpKPC isolates were analyzed, of which 17.1% (7/41) were classified as endemic and 82.9% (34/41) as sporadic. These findings highlight an increase in sporadic isolates following the first COVID-19 case in Brazil, with sporadic isolates comprising 82.9% of the total isolates during this period, compared to 61.5% in the earlier period.

The 40 KpKPC isolates subjected to WGS were as follows: 26 isolates (65%) from rectal surveillance swabs, 11 from tracheal secretions (27.5%), two from blood cultures (5%), and one from bronchoalveolar lavage (2.5%) ( Table 1 ). The distribution of the 40 isolates at the endemic level is shown in Figure 2G (highlighted in red).

**Table 1:** Epidemiological and molecular characteristics of the 40 KpKPC isolates sequenced in this study

Isolate	Date of isolation, month/year	Origin of specimen	MLST type (ST)	Clonal group	Plasmid type	Yersiniabactin/clb	K-Locus	O-Locus
KP728	08/2020	Surveillance rectal swabs	ST437	258	pKPC_FCF_3SP	ICEKp10/clb3	KL36	O4
KP737	07/2020	Surveillance rectal swabs	ST11	258	pKpQIL	ICEKp10/clb3	KL64	O2
KP738	07/2020	Tracheal secretion	ST437	258	pKPC_FCF_3SP	ICEKp10/clb3	KL36	O4
KP801	08/2020	Surveillance rectal swabs	ST11	258	Not detected	ICEKp3	KL15	O4
KP862	08/2020	Surveillance rectal swabs	ST16	Not applicable	pKPC_FCF_3SP	Not detected	KL51	O3b
KP863	08/2020	Surveillance rectal swabs	ST11	258	Not detected	ICEKp4	KL105	O2
KP864	08/2020	Surveillance rectal swabs	ST443	Not applicable	pKpQIL	Unknown	KL146	Unknown
KP867	09/2020	Surveillance rectal swabs	ST307	Not applicable	Not detected	Not detected	KL102	O2
KP868	09/2020	Surveillance rectal swabs	ST13	Not applicable	pKPC_FCF_3SP	ICEKp4	KL3	O1
KP869	09/2020	Surveillance rectal swabs	ST437	258	pKPC_FCF_3SP	Not detected	KL36	O4
KP871	10/2020	Surveillance rectal swabs	ST11	258	Not detected	ICEKp3	KL15	O4
KP872	12/2020	Tracheal secretion	ST11	258	Not detected	ICEKp10/clb3	KL64	O2
KP874	10/2020	Surveillance rectal swabs	ST11	258	Not detected	ICEKp3	KL15	O4
KP875	10/2020	Surveillance rectal swabs	ST11	258	Not detected	ICEKp3	KL15	O4
KP876	11/2020	Surveillance rectal swabs	ST437	258	pKPC_FCF_3SP	Not detected	KL36	O4
KP877	11/2020	Surveillance rectal swabs	ST11	258	pKPC_FCF_3SP	ICEKp3	KL15	O4
KP879	11/2020	Surveillance rectal swabs	ST11	258	Not detected	ICEKp4	KL105	Unknown
KP881	11/2020	Surveillance rectal swabs	ST11	258	pKPC_FCF_3SP	ICEKp10/clb3	KL64	O2
KP882	11/2020	Surveillance rectal swabs	ST11	258	Not detected	unknown	KL64	O4
KP884	11/2020	Surveillance rectal swabs	ST11	258	Not detected	ICEKp10/clb3	KL64	O2
KP885	01/2021	Tracheal secretion	ST16	Not applicable	pKpQIL	ICEKp10/clb3	KL51	O3b
KP886	07/2020	Tracheal secretion	ST512	258	pKpQIL	ICEKp4	KL107	O2
KP887	07/2020	Tracheal secretion	ST437	258	pKPC_FCF_3SP	ICEKp10/clb3	KL36	O4
KP888	08/2020	Tracheal secretion	ST11	258	pKPC_FCF_3SP	ICEKp3	KL15	Unknown
KP889	10/2020	Blood	ST11	258	Not detected	ICEKp3	KL15	Unknown
KP890	11/2020	Tracheal secretion	ST11	258	Not detected	ICEKp3	KL15	O4
KP891	11/2020	Tracheal secretion	ST101	101	Not detected	ICEKp3	KL17	O1
KP896	12/2020	Surveillance rectal swabs	ST11	258	Not detected	ICEKp3	KL15	O4
KP897	12/2020	Surveillance rectal swabs	ST323	Not applicable	Not detected	Not detected	KL21	Unknown
KP898	01/2021	Surveillance rectal swabs	ST307	Not applicable	Not detected	Not detected	KL102	O2
KP899	01/2021	Surveillance rectal swabs	ST11	258	pKPC_FCF_3SP	ICEKp10/clb3	KL64	O2
KP901	01/2020	Surveillance rectal swabs	ST16	Not applicable	pKpQIL	ICEKp10/clb3	KL38	O2
KP903	07/2020	Surveillance rectal swabs	ST11	258	Not detected	ICEKp10/clb3	KL15	Unknown
KP904	11/2018	Surveillance rectal swabs	ST11	258	Not detected	ICEKp3	KL15	O1
KP905	11/2019	Surveillance rectal swabs	ST101	101	Not detected	ICEKp3	KL36	O4
KP906	05/2018	Tracheal secretion	ST437	258	pKPC_FCF_3SP	ICEKp10/clb3	KL36	O4
KP908	05/2018	Bronchoalveolar lavage	ST437	258	pKPC_FCF_3SP	ICEKp10/clb3	KL36	O4
KP910	12/2018	Blood	ST11	258	pKPC_FCF_3SP	ICEKp3	KL15	O4
KP912	11/2019	Tracheal secretion	ST11	258	pKpQIL	ICEKp3	KL15	O4
KP914	10/2019	Tracheal secretion	ST443	Not applicable	pKpQIL	unknown	KL146	O1

KpKPC: *Klebsiella pneumoniae* carrying *bla*_KPC_ gene. MLST: multilocus sequence typing. ST: sequence type.

Multilocus sequence typing identified nine STs among the 40 sequenced KpKPC isolates, with ST11 being the most prevalent (21/40; 52.5%) ( Figure 3B ). During the periods exceeding the upper control limit (July 2020 and January 2021), four distinct STs were observed in July 2020 (ST11, ST437, ST443, and ST512), whereas only three STs (ST11, ST16, and ST307) were detected in January 2021. Before the first reported case of COVID-19 in Brazil (February 25, 2020), the detected STs included ST11, ST16, ST101, ST437, and ST443. After the first reported COVID-19 case, a greater diversity of STs was identified, including ST11, ST13, ST16, ST101, ST307, ST323, ST437, ST443, and ST512 ( Figure 3B and 3C). All STs present before the first COVID-19 case were also identified afterward, but ST13, ST307, ST323, and ST512 were exclusively detected post-COVID-19. Throughout both periods (pre- and post-COVID-19 in Brazil), ST11 predominated, accounting for 37.5% (3/8) of the isolates before the first case and 56.3% (18/32) of the isolates afterward ( Figure 3C ).

The genetic relatedness of the 40 isolates, inferred from cgMLST ( Figure 3D ), revealed high variability in the allelic profiles of cgMLST targets. The observed allelic differences ranged from 17 to 1,852 alleles. These findings were consistent with the PFGE typing results, indicating that the isolates reported during the COVID-19 period at the evaluated hospital could be classified as sporadic. No outbreak was detected during the study period, based on the threshold of ≤10 allelic differences as suggested in the literature.^( 35 )^

**Figure 4:**
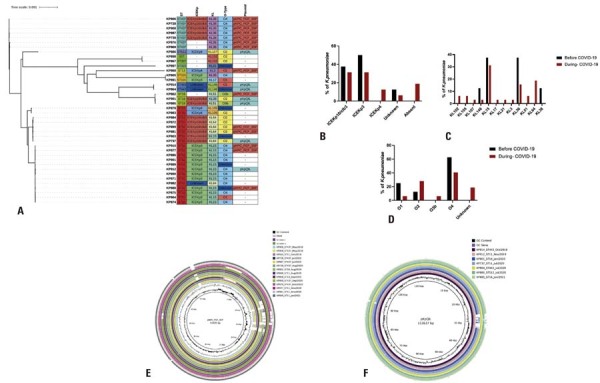
(A) Phylogenetic tree based on core genome alignment. ST: Sequence type; ICEKp: integrative and conjugative element of *Klebsiella pneumoniae* ; KL: Capsule synthesis loci; O-type: O-specific polysaccharides (lipopolysaccharide); Plasmid: Presence or absence of plasmids previously reported in isolates from the same hospital. (-): Isolates negative for the characteristic sought. (B) Distribution of ICEKp. (C) KL type. (D) O-type among the *K. pneumoniae* carrying *bla* KPC (KpKPC) isolates subjected to whole genome sequencing. (E) Comparison using the plasmid pKPC_FCF_3SP (accession number: CP004367.2) as a reference, a plasmid identified in 14 isolates with highly similar plasmids. (F) Comparison using the plasmid pKpQIL (accession number: GU595196.1) as a reference, a plasmid identified in seven isolates that contained highly identical plasmids.

Core genome analysis of the 40 KpKPC isolates identified 3,936 genes as part of the core genome (genes shared by 99–100% of the isolates), while the pan-genome comprised 10,575 genes. A phylogenetic tree was generated from the core genome alignment using Roary software ( Figure 4A ). This tree shows that the isolates were grouped by ST, with high similarity observed among isolates within each ST. The ST11 KpKPC isolates formed three main clusters, which were further subdivided based on the type of ICEKp identified in each isolate ( Figure 4A ).

### Virulome, plasmidome, and resistome analysis

Virulence factor typing revealed that nearly all isolates (34/40 KpKPC, 85%) carried ICEKp, and a combination of ICEKp and colibactin was present in 13 KpKPC isolates (32.5%) ( Table 1 and Figure 4A ). ICEKp4 was identified only in isolates that appeared after the first case of COVID-19, and a higher proportion of isolates carrying ICEKp3 was also observed during this period ( Figure 4B ). Both K-typing and O-typing have significant clinical and epidemiological relevance. Twelve K-types were identified, with KL15 and KL36 being the most prevalent, occurring in 32.5% and 20% of the sequenced KpKPC populations, respectively. However, only three KL types were found in both periods (before and during the COVID-19 pandemic): eight KL types were exclusively identified during the pandemic, and KL38 was found exclusively before the pandemic ( Figure 4C ). Four known O-types were identified, with O4 being the most prevalent and observed in 18 isolates (45%) ( Table 1 and Figure 4D ). Among the four identified O-types, only O3b was detected exclusively during the COVID-19 period ( Figure 4D ).

Regarding plasmid replicon typing, 23 plasmid replicons were identified among the 40 KpKPC isolates. IncFIB (K) was the most prevalent replicon, detected in 80% (32/40) of KpKPC isolates. More than 50% (12/23) of the replicons were found in at least one KpKPC isolate, both before and after the first reported COVID-19 case, whereas approximately 47% (11/23) were identified exclusively in isolates collected during the pandemic (Figure 1S, Supplementary Material ). We also evaluated whether the plasmids were similar to those reported previously at the same hospital.^( 9 )^ Fourteen KpKPC isolates (35%) harbored a plasmid highly similar to the previously reported pKPC_FCF3SP plasmid (IncN) (accession number: CP004367.2), and seven isolates (17.5%) carried a plasmid highly similar to the previously reported pKpQIL plasmid (IncFII) (accession number: GU595196.1) ( Figure 4E and 4F ). A higher proportion of isolates carrying the pKpQIL plasmid was identified before the first reported case of COVID-19 in Brazil (37.5% *versus* 12.9%) (Figure 2S, Supplementary Material ).

Antimicrobial resistance genes associated with 12 classes of antimicrobials were identified (Table 1S, Supplementary Material ). Except for the *bla*_KPC_ gene, the presence of which was confirmed by real-time PCR and which was part of the selection criteria for this study, no resistance genes were detected in all isolates. The most frequently reported genes before and during the COVID-19 pandemic were *fosA6* (fosfomycin resistance glutathione transferase fosA6) (6/8; 75% *versus* 29/32; 90.6%), *oqxA1* (multidrug efflux RND transporter periplasmic adaptor subunit oqxA) (5/8; 62.5% *versus* 24/32; 75%), *bla*_SHV-158_(class A beta-lactamase SHV-158) (5/8; 62.5% *versus* 24/32; 75%), *oqxB* (multidrug efflux RND transporter permease subunit oqxB) (5/8; 62.5% *versus* 22/32; 68.8%), and *sul1* (sulfonamide-resistant dihydropteroate synthase sul1) (4/8; 50% *versus* 21/32; 65.6%) (Table 1S, Supplementary Material ). The *bla*_OXA-β_-lactamase gene (class D) was detected in 55% (22/40) of KpKPC isolates and was more prevalent before COVID-19 (6/8; 75%) than during the pandemic (16/32; 50%). CTX-M enzyme-related genes were also analyzed, and the *bla*_CTX-M-15_ (extended-spectrum class A beta-lactamase CTX-M-15) gene was detected in 50% (20/40) of isolates, with a higher proportion before COVID-19 (6/8; 75%) than during the pandemic (14/32; 43.8%). The TEM-1 β-lactamase (broad-spectrum class A beta-lactamase TEM-1) gene was identified in 45% (18/40) of KpKPC isolates, with similar proportions between the two periods (50% and 43% before and during the COVID-19 pandemic, respectively). Approximately 43% (26/60) of the AMR genes were identified exclusively in isolates reported after the first case of COVID-19 (Table 1S, Supplementary Material ).

## DISCUSSION

This study examined the potential impact of the COVID-19 pandemic on the population structure, antimicrobial resistance patterns, and virulence gene profiles of KPC-producing *K. pneumoniae* isolates from a tertiary care hospital in Brazil. The isolates corresponded to the pre- and during-COVID-19 periods, with the first COVID-19 case detected in Brazil on February 25, 2020.^( 36 )^

Secondary bacterial infections associated with severe outcomes in COVID-19 patients have been widely reported.^( 13 , 37 )^ A high incidence of Gram-negative infections, especially with *K. pneumoniae* , has been linked to poorer COVID-19 outcomes.^( 13 , 38 , 39 )^ Additionally, increased antibiotic consumption likely accelerated resistance in multidrug-resistant strains, such as pathogens from the ESKAPE group.^( 40 , 41 )^ Managing *K. pneumoniae* infections has become critical due to the pathogen’s resistance to antimicrobials, including β-lactams, aminoglycosides, quinolones, and polymyxins.^( 36 )^

Recently, a review of carbapenem-resistant *K. pneumoniae* infections in patients hospitalized for COVID-19 (11 studies and 6 countries: Italy, China, Egypt, United States, Spain, and Peru) showed that the prevalence ranged from 0.4% to 53%. The most commonly associated β-lactamases were KPC, OXA-48, and NDM.^( 4 )^ A Mexican study reported increased carbapenem resistance in *K. pneumoniae* blood isolates, from 7.3% (2019) to 14.6% (2020).^( 42 )^ In Brazil, *bla*_KPC_ and *bla*_NDM_ detection rates in Enterobacterales increased from 57.1% to 61.8% and 18.7% to 28.0%, respectively, during the pandemic.^( 43 )^ In our study, carbapenem resistance increased from 32.5% (2019) to 60.3% (2020), remaining stable until January 2021.

The *bla*_KPC_ gene is the main mechanism driving carbapenem resistance in Brazil^( 44 )^ and in this hospital.^( 9 , 11 )^ KPC-2–producing *K. pneumoniae* isolates are endemic to Brazil and mostly belong to CC258, particularly ST437, ST258 (clade II), and ST11.^( 10 , 12 )^ In this study, KPC was detected in all 40 sequenced strains, predominantly from ST11 and ST437 lineages (70%). PFGE analysis classified 75% (21/28) of ST11 and ST437 strains as sporadic, consistent with this and previous work by our group,^( 11 )^ whereas WGS revealed diverse genetic features, including four ICEKp types (ICEKp4, ICEKp3, and ICEKp10+clb), associated with three ST11 clusters.

Mobile genetic elements facilitate *bla*_KPC_ dissemination. Over 52.5% (21/40) of the strains carried plasmids resembling pKPC_FCF3SP (IncN) or pKpQIL-like plasmids, as well as IncFII plasmids found in our hospital.^( 9 , 45 )^ The prevalence of pKpQIL-like plasmids decreased post-pandemic, while plasmid-negative strains increased by 25%, possibly reflecting hospital transfers of colonized patients.^( 9 )^

Multidrug resistance was widespread, consistent with other studies.^( 46 )^ Aminoglycosides (amikacin and gentamicin) remained effective overall; however, resistance increased. Ten aminoglycoside resistance genes were identified, with *aac(6′)-Ib-D181Y* being the most frequent.^( 46 )^

An observational retrospective study conducted in São Paulo State, Brazil, identified carbapenem-resistant *K. pneumoniae* isolates in 62% (153/246) of ICU patients between January 2018 and July 2020.^( 47 )^ In our study, carbapenem-resistant *K. pneumoniae* accounted for 48.3% of the *K. pneumoniae* isolates, which is lower than the rate reported previously. Gaspar et al.^( 47 )^ observed that the incidence of healthcare-associated infections associated with carbapenem-resistant *K. pneumoniae* increased during the pandemic. We observed an increase in the incidence of carbapenem-resistant *K. pneumoniae* per 10,000 patient-days after the first COVID-19 report in Brazil compared with the pre-COVID-19 period. Notably, when the HI rate for carbapenem-resistant *K. pneumoniae* exceeded the upper control limit during both the COVID-19 pandemic and pre-pandemic periods, PFGE and MLST classified the isolates as sporadic. This finding is supported not only by this study but also by previous reports from the same hospital.^( 9 , 11 )^

Virulence factors, including yersiniabactin (mobilized by ICEs), are widely distributed.^( 13 , 48 )^ Yersiniabactin is mobilized by an ICE. In the present study, four types of ICEKp (ICEKp3, ICEKp4, ICEKp10+clb, and unknown) were identified. ICEKp4 was detected only during the pandemic in the clinical ward. Nine main O-antigen clusters have been described for *K. pneumoniae* , and serotypes O1, O2, and O3 are associated with almost 80% of infections.^( 49 , 50 )^ Here, four O-antigen types (O1, O2, O3b, O4) were identified, with O4 and O2 predominating during the pandemic and O3b found only during the pandemic.

This study provides insights into *K. pneumoniae* epidemiology and highlights the broad challenges associated with antimicrobial resistance during the COVID-19 pandemic. However, molecular analysis was limited to isolates collected during routine procedures by the clinical laboratory team and infection control service. Because there was no sampling scheme dedicated to this study, the included isolates may not completely represent the diversity of *K. pneumoniae* clones, antimicrobial resistance patterns, and virulence profiles. Additionally, we focused on analyzing KPC-producing *K. pneumoniae* because of its clinical relevance and high prevalence in this clinical setting. A more extensive study design could provide more insights into the *K. pneumoniae* population and the dissemination of resistance and virulence-related genes.

## CONCLUSION

This study highlighted the genetic diversity, antimicrobial resistance, and virulence factors of *K. pneumoniae* isolates before and during the COVID-19 pandemic. The increase in carbapenem-resistant *K. pneumoniae* during the pandemic underscores the impact of increased antibiotic use. The predominance of the *bla*_KPC_ gene and the role of mobile genetic elements, such as plasmids and ICEKp types, emphasize the need for enhanced surveillance and stewardship strategies. Shifts in O-antigen types and the emergence of ICEKp4 during the pandemic reflect the adaptive evolution of *K. pneumoniae* in response to clinical pressure. In intensive care unit settings, targeted interventions, including robust infection control, prudent antimicrobial use, and continuous genomic surveillance, are crucial for mitigating the spread of resistant strains.

## SUPPLEMENTARY MATERIAL

Figure 1SDistribution of replicons during the studied periods
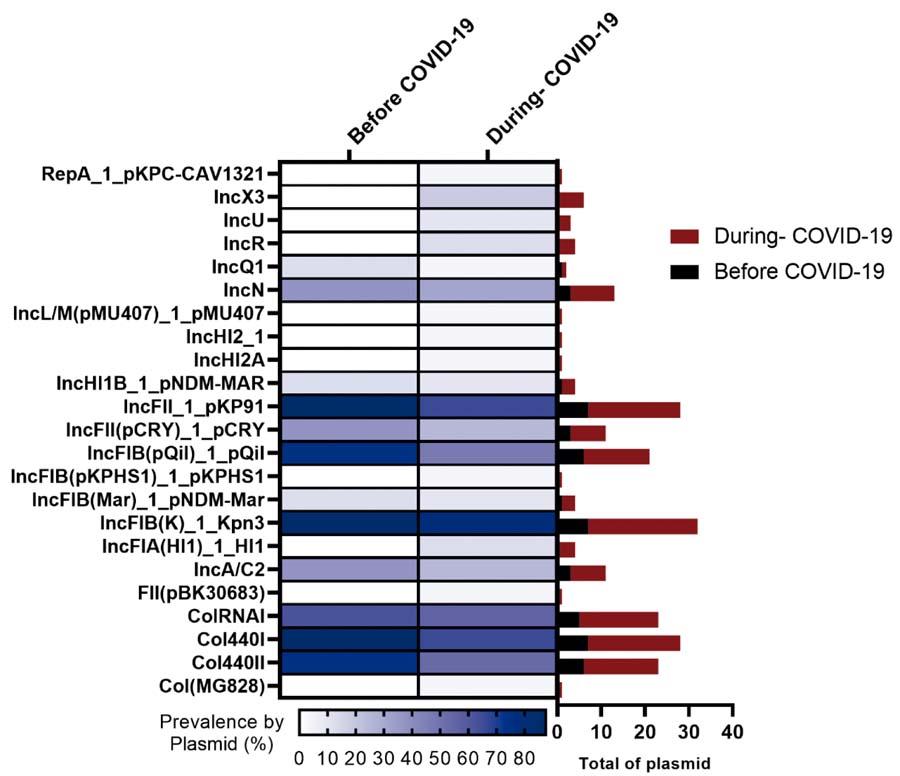
COVID-19: coronavirus disease 2019.

Figure 2SProportion of isolates carrying pKPC_FCF3SP and pKpQIL plasmids before and during the COVID-19 pandemic
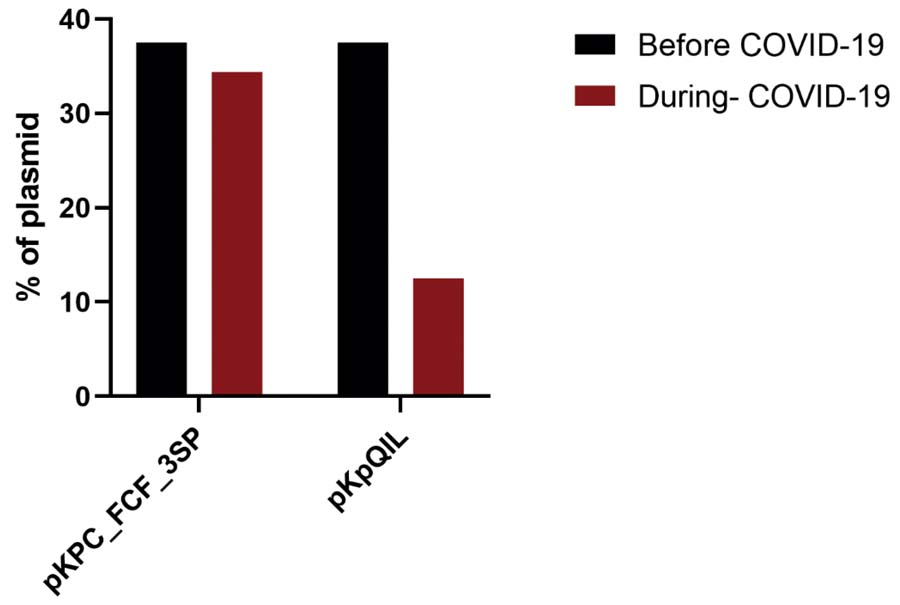
COVID-19: coronavirus disease 2019.

Table 1S Proportion of antimicrobial resistance genes

**Table:** 

Gene	Before COVID-19	During-COVID-19	Before COVID-19	During- COVID-19	Class
aac(3)-IId	25.00	15.63	1	1	Aminoglycosides
aac(3)-IIe	50.00	40.63	1	1	Aminoglycosides
aac(6′)-Ib-AKT	0.00	21.88	0	1	Aminoglycosides
aac(6′)-Ib-D181Y	75.00	50.00	1	1	Aminoglycosides
aadA1	0.00	3.13	0	1	Aminoglycosides
aadA2	50.00	34.38	1	1	Aminoglycosides
aph(3′)-Ia	37.50	34.38	1	1	Aminoglycosides
aph(3″)-Ib	0.00	15.63	0	1	Aminoglycosides
aph(3′)-VIa	12.50	3.13	1	1	Aminoglycosides
aph(6)-Id	0.00	12.50	0	1	Aminoglycosides
blaCTX-M-15	75.00	43.75	1	1	Beta-Lactam
blaCTX-M-2	0.00	3.13	0	1	Beta-Lactam
blaKPC-2	87.50	71.88	1	1	Beta-Lactam
blaKPC-3	12.50	6.25	1	1	Beta-Lactam
blaKPC-33	0.00	3.13	0	1	Beta-Lactam
blaLAP-2	37.50	21.88	1	1	Beta-Lactam
blaNDM-1	0.00	6.25	0	1	Beta-Lactam
blaNDM-7	0.00	3.13	0	1	Beta-Lactam
blaOXA-1	75.00	50.00	1	1	Beta-Lactam
blaOXA-2	0.00	15.63	0	1	Beta-Lactam
blaOXA-9	25.00	6.25	1	1	Beta-Lactam
blaSHV-101	0.00	3.13	0	1	Beta-Lactam
blaSHV-106	0.00	6.25	0	1	Beta-Lactam
blaSHV-145	25.00	9.38	1	1	Beta-Lactam
blaSHV-158	62.50	75.00	1	1	Beta-Lactam
blaSHV-187	0.00	3.13	0	1	Beta-Lactam
blaSHV-212	12.50	3.13	1	1	Beta-Lactam
blaTEM-1	50.00	43.75	1	1	Beta-Lactam
bleMBL	0.00	9.38	0	1	Bleomycin
catA1	0.00	15.63	0	1	Chloramphenicol
catA2	12.50	3.13	1	1	Chloramphenicol
dfrA12	50.00	31.25	1	1	Trimethoprim
dfrA14	12.50	18.75	1	1	Trimethoprim
dfrA26	0.00	3.13	0	1	Trimethoprim
dfrA30	25.00	12.50	1	1	Trimethoprim
dfrA32	0.00	3.13	0	1	Trimethoprim
ere(A)	0.00	3.13	0	1	Erythromycin
fosA_gene	12.50	3.13	1	1	Fosfomycin
fosA5	12.50	6.25	1	1	Fosfomycin
fosA6	75.00	90.63	1	1	Fosfomycin
fosA7.4	0.00	3.13	0	1	Fosfomycin
mph(A)	75.00	53.13	1	1	Macrolide
oqxA	62.50	75.00	1	1	Quinolone
oqxA10	25.00	9.38	1	1	Quinolone
oqxA5	0.00	9.38	0	1	Quinolone
oqxA6	0.00	3.13	0	1	Quinolone
oqxB	62.50	68.75	1	1	Quinolone
oqxB12	0.00	3.13	0	1	Quinolone
oqxB19	0.00	9.38	0	1	Quinolone
oqxB31	12.50	3.13	1	1	Quinolone
oqxB32	12.50	6.25	1	1	Quinolone
qnrB1	0.00	15.63	0	1	Quinolone
qnrE1	0.00	3.13	0	1	Quinolone
qnrS1	25.00	25.00	1	1	Quinolone
sat2_fam	0.00	12.50	0	1	Streptothricin
sul1	50.00	65.63	1	1	Sulfonamide
sul2	37.50	43.75	1	1	Sulfonamide
tet(A)	37.50	15.63	1	1	Tetracycline
tet(B)	0.00	3.13	0	1	Tetracycline
tet(D)	25.00	18.75	1	1	Tetracycline

COVID-19: coronavirus disease 2019.
